# Short-term alteration of biotic and abiotic components of the pelagic system in a shallow bay produced by a strong natural hypoxia event

**DOI:** 10.1371/journal.pone.0179023

**Published:** 2017-07-17

**Authors:** Eduardo Hernández-Miranda, Rodrigo Veas, Valeria Anabalón, Renato A. Quiñones

**Affiliations:** 1 Programa de Investigación Marina de Excelencia (PIMEX), Facultad de Ciencias Naturales y Oceanográficas, Universidad de Concepción, Concepción, Chile; 2 Interdisciplinary Center for Aquaculture Research (INCAR), Casilla 160-C, Universidad de Concepción, Concepción, Chile; 3 Department of Oceanography, Casilla 160-C, Universidad de Concepción, Concepción, Chile; University of Waikato, NEW ZEALAND

## Abstract

In January 2008 there was an intensive and extensive upwelling event in the southern Humboldt Current System. This event produced an intrusion of water with low dissolved oxygen into Coliumo Bay, which caused massive mortality and the beaching of pelagic and benthic organisms, including zooplankton. During this event, which lasted 3 to 5 days, we studied and evaluated the effect of the hypoxic water in the bay on the abundance of macrozooplankton, nanoplankton and microphytoplankton, the concentration of several nutrients and hydrographic conditions. At the beginning of the hypoxia event the water column had very low dissolved oxygen concentrations (<0.5 mL O_2_ L^-1^), low temperatures and high salinity which are characteristics of the oxygen minimum zone from the Humboldt Current System. Redox, pH, nitrate, phosphate, silicate and chlorophyll-*a* values were the lowest, while nitrate and the phaeopigment values were the highest. The N:P ratio was below 16, and the abundance of nano- and microphytoplankton were at their lowest, the latter also with the lowest proportion of live organisms. Macrozooplankton had the greatest abundance during hypoxia, dominated mainly by crustacean, fish eggs and amphipods. The hypoxia event generated a strong short-term alteration of all biotic and abiotic components of the pelagic system in Coliumo Bay and the neighboring coastal zone. These negative effects associated with strong natural hypoxia events could have important consequences for the productivity and ecosystem functioning of the coastal zone of the Humboldt Current System if, as suggested by several models, winds favorable to upwelling should increase due to climate change. The effects of natural hypoxia in this coastal zone can be dramatic especially for pelagic and benthic species not adapted to endure conditions of low dissolved oxygen.

## Introduction

Coastal upwelling in the Humboldt Current System (HCS) off central-south Chile begins with an increase in southwest winds, favoring the arrival of deeper water rich in nutrients and low in dissolved oxygen (*i*.*e*. Equatorial Subsurface Water; ESSW, [1]). Although the fertilizing effect of upwelling on the surface layer of the ocean at a seasonal time scale is well known (*e*.*g*. [2, 3]), the initial stage of the process at a time scale of hours/days has not been well studied. The initial arrival of low-oxygen water in shallow areas has a negative effect on their inhabitants, mainly on species poorly adapted to anaerobic conditions [4–6]. The magnitude of this effect depends on the intensity of the event and the residence time of the low-oxygen water in the coastal zone; it may have benign consequences or generate strong local perturbations in the ecosystem [7–9]. Several studies have proposed that values of dissolved oxygen less than 1.4 mL O_2_ L^-1^ produce negative effects on marine biota, including death and massive stranding (*e*.*g*. [5, 10–13]). The effect of low oxygen conditions on plankton has mostly been studied in oceanic waters (*e*.*g*. [14]) and from the middle to the outer continental shelf *(e*.*g*. [15]), whereas the effect on ecosystems from the interior continental shelf has not been well studied, with the exception of the dead zones associated with the discharge of large rivers (*e*.*g*. [16]), hypoxic events in estuaries (*e*.*g*. [17]) and hypoxia related to anthropogenic causes [13]. Rabalais et al. [13] proposed a conceptual model that relates the spatial and temporal scales of hypoxic events to the relative influence of human activity and natural processes. In this model temporal events of days to weeks tend to be related to small-scale spatial systems (small tributaries, rivers-estuaries and fjords), with causes strongly related to human activity. In this study we describe the effect of a strong natural hypoxia event caused by coastal upwelling that occurred at a temporal scale of days in a small, shallow bay of the HCS.

**Fig 1:**
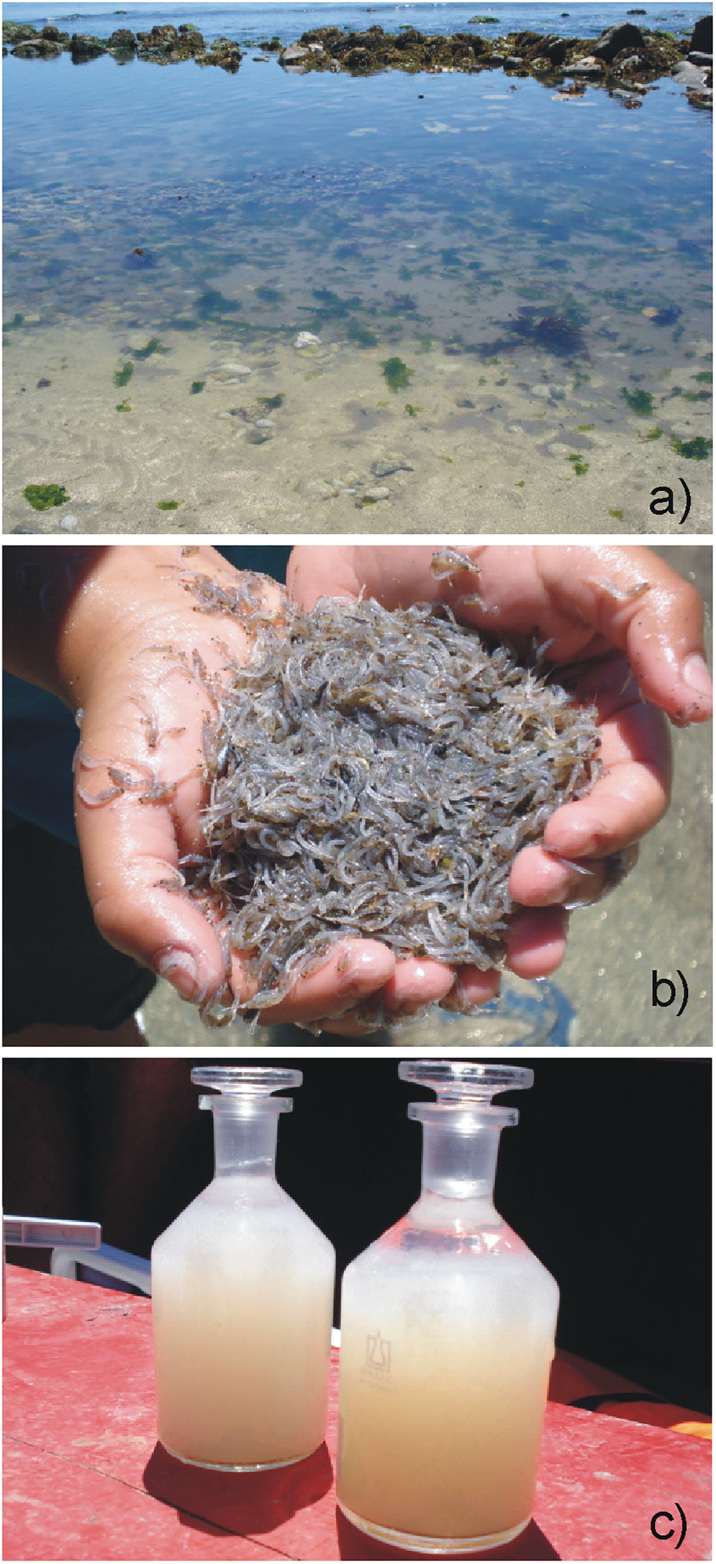
Photographs of the intertidal zone facing site E4 in Coliumo Bay. (a, b) The grey patches are thousands of beached and dead individuals of Miscidaceae (*Neomysis* sp.). Also note the whitish color of the bottle to measure dissolved oxygen mixed with the chemicals for the Winkler method analysis (c).

Off the south-central coast of Chile in the first days of January 2008, there was an intense and extensive upwelling event that involved a spatial scale of hundreds of kilometers. This produced an intrusion into the coastal area of ESSW from the oxygen minimum zone (OMZ), and produced massive mortality and beaching of fish, crustaceans and molluscs in Coliumo Bay [18–21], as well as zooplankton (see Fig 1). The OMZ in the Humboldt Current System is characterized by an oxygen concentration of <20 μmol kg^−1^ (0.5 mL O_2_ L^-1^) (*e*.*g*. [22–25]). This raises the question of what were the immediate effects of, and the dynamic response to the entrance of this hypoxic water on the hydrography, nutrients and plankton of Coliumo Bay. Our hypothesis was that natural hypoxia events in shallow coastal zones would produce a strong perturbation of the neritic ecosystem. However, the final ecosystem response depends on the spatial scale affected by hypoxia and the duration of the event. Given the intense water exchange of Coliumo Bay with the open ocean, the negative effects may have been absorbed rapidly, which would have been observed as a short-term succession of species and environmental conditions, with recovery in a few days. This can be evaluated by following the hypoxic event over a short time scale (*i*.*e*. days), using information about the hydrographic characteristics of the water column and plankton abundance and composition in Coliumo Bay. Thus, the objectives of this study were to quantify the dynamics of the neritic ecosystem during and after this natural strong hypoxia event using information on (i) hydrographic variables (ii) nutrients concentration, (iii) abundances of the main taxa of nanoplankton (iv) the abundances of the main taxa of microphytoplankton, and (v) abundances of the main groups of macrozooplankton.

## Material and methods

### Ethics statement

Coliumo bay is a free access marine area and no specific permits were required to conduct sampling activities. The oceanographic sampling was authorized by the Hydrographic and Oceanographic Service of the Chilean Navy (SHOA; ORDINARIO N°13270/24/238/Vrs). The field studies did not involve endangered or protected species.

### Sampling design and laboratory analysis

**Fig 2:**
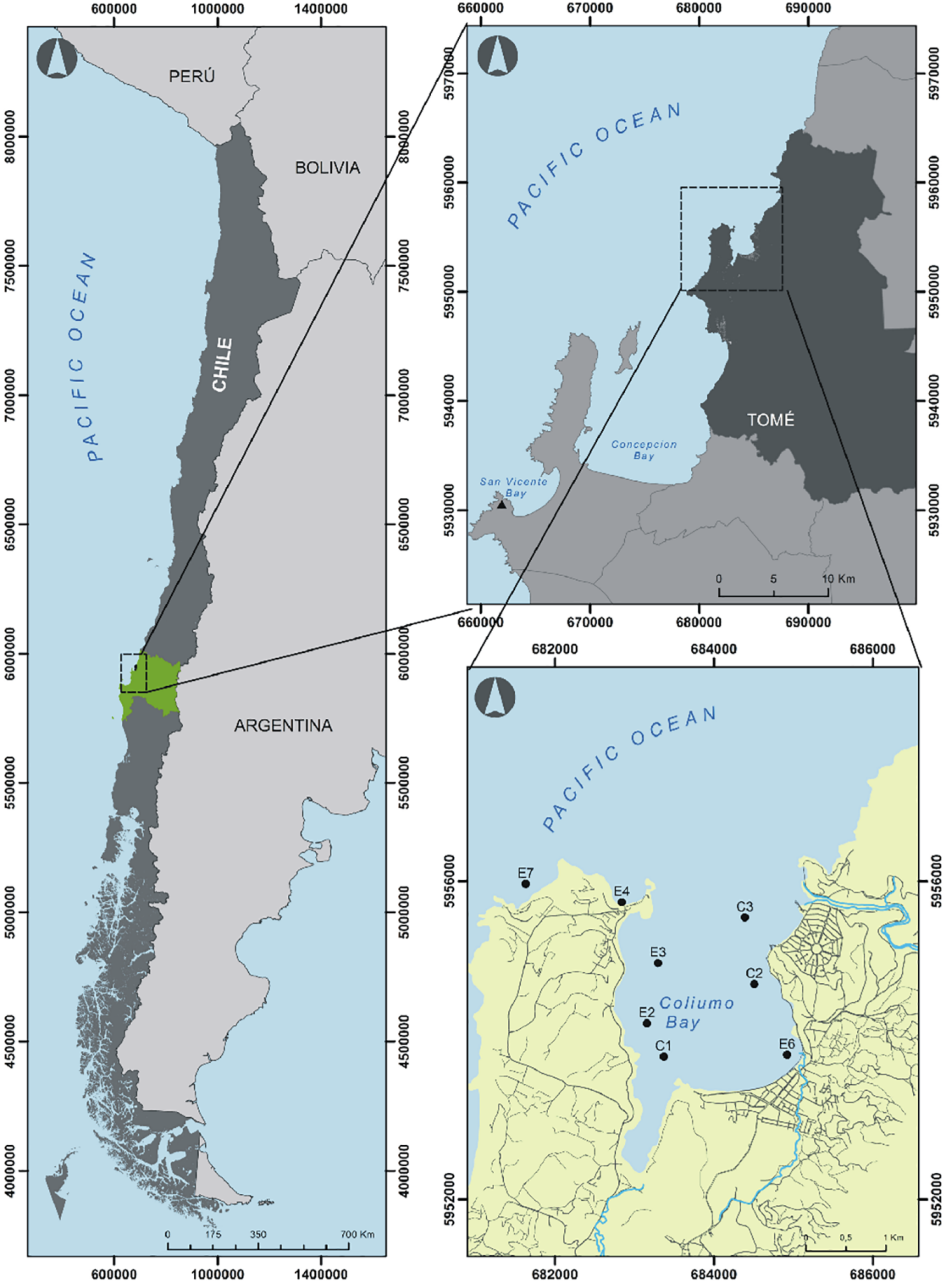
Coliumo Bay study area and sampling sites. E: sites sampled during the hypoxia event. C: sites sampled seasonally from 2007 to 2009. The black triangle in San Vicente Bay indicates the location of the wind measurement station. E6: Site where sub-tidal temperature was recorded with data loggers.

Herein, we use the term hypoxia as those environmental conditions where the dissolved oxygen level in the water column is less than 1.4 mL O_2_ L^-1^, which is a threshold known to produce negative effects on marine biota (*e*.*g*. [5, 10–13]). Coliumo bay is a shallow embayment no more than 25 m deep at its mouth, which is located to the north (Fig 2) and it covers an area of approximately 6 km^2^ (36°32.767°S; 72°57.113°W). During the hypoxia event with massive mortality of organisms [15], we sampled the bay on five separate occasions between January 3^rd^, and 18^th^, 2008, (Jan. 3^rd^, 4^th^, 7^th^, 9^th^, and 18^th^). At each of the sampling sites within (sites E2, E3, E6) and outside the bay (sites E4 and E7), we measured nutrients (nitrate, nitrite, phosphate and silicic acid), chlorophyll-*a*, phaeopigments, nanoplankton, microphytoplankton, macrozooplankton, and hydrographic variables of the water column (temperature, salinity, dissolved oxygen) (Figs 1 and 2).

To characterize the hydrography of Coliumo Bay, vertical profiles for salinity, temperature (°C) and dissolved oxygen (mL O_2_ L^-1^) were taken at the sampling sites, measured with a CTD-O (SAIV A/S, model SD204). We also recorded pH and redox (mV) with a multi-parameter sensor YSI-556MPS at the surface and the bottom of the water column. The dissolved oxygen concentration was calibrated with a semi-automatic version of the Winkler method [26] as modified by [27]. The calibration was based on a photometric end-point detector, a Dosimat 665 (Metrohom) and a chart recorder.

We also obtained a time series of continuous records of the subsurface sea temperature, tide height, winds and satellite images of sea surface temperature (SST). Subsurface temperature was measured with thermistors (HOBO Data Loggers UTBI-001 TidbiTv2) installed in Coliumo Bay at a depth of 3m (Fig 2), set to record data every 10 minutes. Data on tide heights (m) were obtained from the Hydrographic and Oceanographic Service of the Chilean Navy (SHOA: www.shoa.cl). The wind data time series was collected at Faro Hualpén (36°45'25'' S, 73°11'51'' W) field station (Fig 2). Wind magnitude and direction were recorded hourly. Both wind and subsurface sea temperature data were represented using smoothed average values. Satellite images of SST (°C) were obtained from the ANTARES Observation Network (http://antares.ws).

Seawater samples for nutrient analysis were collected at the surface and the sea bottom using a Niskin bottle; 120 ml of seawater from each sample was filtered through a 0.7 μm pore Millipore glass fiber filter, 25 mm in diameter, to eliminate particulate material. The remaining water sample was frozen until laboratory analysis. Nitrate (μM) was determined by the spectrophotometric method using a cadmium sponge [28]; nitrite (μM) using spectrophotometry [29], and phosphate (μM) and silicic acid (μM) were determined by the method described in [30]. In addition, at each sampling site 500 mL of surface and bottom seawater were filtered using GF/F filters (0.7 μm), then frozen (-20°C) and kept in the dark for later chlorophyll *a* and phaeopigment determination in the laboratory (mg/m^3^). Determinations were made using a Turner Designs fluorimeter following the methods described in [31].

Subsurface nano- and microphytoplankton samples were collected using 5-L Niskin bottles and immediately concentrated and preserved in a glass bottle (250 mL) with an acid solution of iodine (2% final concentration). The nano- and microphytoplankton samples were analyzed with an inverted microscope (Nikon Eclipse TE2000S), following the Ütermohl method [32] with 1000x resolution. Counts were made until at least 200 cells of the dominant taxa per sample were recorded in the nanoplankton and until at least 100 cells of the dominant taxa per sample in the microphytoplankton composition. Cell counts were classified into live and dead; live cells were defined as those with intact cytoplasm, while dead cells included frustules, thecae, coccoliths and empty shells. Cells were grouped into diatoms, dinoflagellates, ciliates, cocolithophorids and cyanobacteria. The following guides were used for the taxonomic identification [33–42]. Nano- and microphytoplankton abundances were expressed as Cel mL^-1^ (See also S1 Database).

In addition, samples from the nanoplankton fraction were analyzed by epifluorescence to distinguish trophic function (autotrophic or heterotrophic). Water samples were transferred to 20 and 50-mL tubes and immediately preserved with glutaraldehyde (2% final concentration) and stored under cold (~4°C) and dark conditions. After a few days, these samples were stained with a mixture of Proflavine (3–6 diamino-acridine hemi-sulfate) and DAPI (4, 6-diamidino-2-phenylindole) at a final concentration of 0.005% [43], and filtered onto polycarbonate black filters (0.8 μm pore). These filters were mounted on glass slides and stored at -20°C in the dark until subsequent analysis. Nanoplankton was counted at 1600x magnification with an epifluorescence microscope (Zeiss Axioscope Plus 2) equipped with multiple light filters (UV, blue and green). The counting included at least 100 cells of the most dominant taxa from each sample. Cells were grouped as autotrophic and heterotrophic. Nanoplankton abundances are reported as Cel mL^-1^.

Macrozooplankton was collected by oblique tows, using a conical net with a mouth 50 cm in diameter and a mesh size of 500 μm equipped with a calibrated Hydrobios flowmeter. Zooplankton counts were standardized to N° Ind. 100 m^-3^ (See also S1 Database). Tows were conducted between the surface and 5 to 15 m, depending on the depth at each sampling site. Samples were fixed in 96% ethanol, separated, counted and identified in the laboratory using an Olympus SZ-61 stereoscopic microscope. In addition, for comparative purposes, on a longer time scale, we used the data from seasonal/trimestral samples of macrozooplankton that were taken in Coliumo Bay between January 2007 and January 2009 (9 sampling periods in the sites C1, C2 and C3; January, May, August, and November 2007, January, April, July, and October 2008, and January 2009; See Fig 2). These samplings were conducted with a bongo net with a mouth of 60 cm in diameter and a 500 μm mesh size equipped with a calibrated Hydrobios flowmeter. The tows were oblique from the surface to 5–15 m depending on the depth at each sampling site. Zooplankton was preserved, analyzed, and standardized (N° ind 100 m^-3^) similarly to the samples collected during the hypoxia period (See also S1 Database).

### Statistical analyses

Multidimensional scaling analysis for the microphytoplankton (live and dead cells), macrozooplankton and the hydrographic-environmental conditions of the water column were done as follows: (i) microphytoplankton and macrozooplankton assemblages: non-metric multidimensional scaling analysis (nMDS) using the Bray-Curtis dissimilarity resemblance measure with non-transformed data, and Jaccard similarity coefficient with the data converted to presence-absence [44, 45], (ii) hydrographic-environmental variables: multidimensional scaling analysis (MDS) using Euclidean distances with normalized data. PERMANOVA was used to evaluate statistical differences according to the factors “sampling zone” and “period” using resemblance matrices similar to those for the nMDs and MDS analyses. “Sampling zones” correspond to sampling sites inside or outside Coliumo Bay. Because there was no microphytoplankton and hydrographic/environmental sampling before the hypoxia event, the factor “period” covers only data obtained during or after the hypoxia event. In the case of macrozooplankton, the factor “period” included before, during and after the hypoxia event. Accordingly, we used data obtained during the hypoxia event and from the seasonal/trimestral samples collected over three years. In this macrozooplankton analysis (nMDS and PERMANOVA) we used Jaccard similarity coefficient with the aim to minimize the potential effect of capturing higher abundances of organisms with bongo plankton net with a larger diameter. All PERMANOVA analyses used the unrestricted model with 9999 permutations. Multivariate over-dispersion of the data was evaluated with the PERMDISP routine using resemblance matrices similar to those of PERMANOVA. Posteriori *pair-wise* tests were conducted when statistical differences were found in PERMANOVA. Analyses were made with the software PRIMER v6 and PERMANOVA+ for PRIMER [46–48]. Complementary analysis of partial least squares (PLS) was performed for the days of the hypoxia event. We evaluated which hydrographic variables obtained from the water column explained the temporal variability of the live and dead microphytoplankton and total macrozooplankton. The analyses were made with MINITAB v15. Finally, we evaluated the correlation between the abundances of main groups of macrozooplankton and dissolved oxygen concentration using the Spearman rank correlation with all available data (N = 40 samples).

## Results

### Upwelling event

The coastal area influenced by waters from the OMZ (*i*.*e*. ESSW) was about 50,000 km^2^, including *ca*. 500 linear km between approximately 34°00’ and 39°00’ S and up to 74°00’ W (S1 Fig). The upwelling event began on December 31^st^, 2007, when southwest winds intensified, transferring their momentum and friction to the superficial layer of the water column. They continued intermittently until January 25^th^, 2008, (S2 Fig). The transfer of momentum from the sea wind was detected in Coliumo Bay on January 1^st^, 2008, one day after wind intensity increased (S2 Fig). The temperature recorded in the sub-tidal zone of Coliumo Bay (3 m depth) showed a strong decline of about 4°C, coincident with the neap tide, that is, with the lowest monthly amplitude. The temperature remained below 14°C until about January 23^rd^, 2008, increasing in the following days until it reached values close to 16°C, which were those recorded before the event. The temperature increase was a consequence of the decreased intensity of southwest winds (S2 Fig).

### Hydrographic variables

On January 3^rd^, and 4^th^, 2008, (fourth and fifth days of the upwelling event), which were the days of acute hypoxia, dissolved oxygen concentration in the entire water column of the bay were less than 0.5 mL O_2_ L^-1^, while outside the bay they were 1–2 mL O_2_ L^-1^. The lowest temperatures (10.5 to 11.5°C) were found up to 2 m deep inside and outside of the bay. Salinity was over 34.2 at the surface, both inside and outside the bay (S3 Fig). Between January 7^th^, and 9^th^, colder and saltier waters with less dissolved oxygen deepened both inside and outside Coliumo Bay. Most oxygen values were above 2 mL O_2_ L^-1^, attributable to the replacement of water in the bay and the ventilation due to mixing (S3 Fig). Beginning on January 16^th^, there was a second upwelling event; dissolved oxygen decreased again, but with values mostly above 1 mL O_2_ L^-1^, indicating that the hypoxic event was less intense than the previous one (S3 Fig). During the hypoxia event (January 3^rd^, and 4^th^) the lowest pH and redox values were observed. pH fluctuated between 7.47 and 7.56 and the redox was between -31 to 40 (bottom) and 55 to 90 (surface). Under non-upwelling conditions (January 7^th^, and 9^th^) pH was between 7.70 and 8.00, while redox was between 40 and 250 (bottom) and 62 to 277 (surface).

### Nutrients and biological variables dynamics

**Fig 3:**
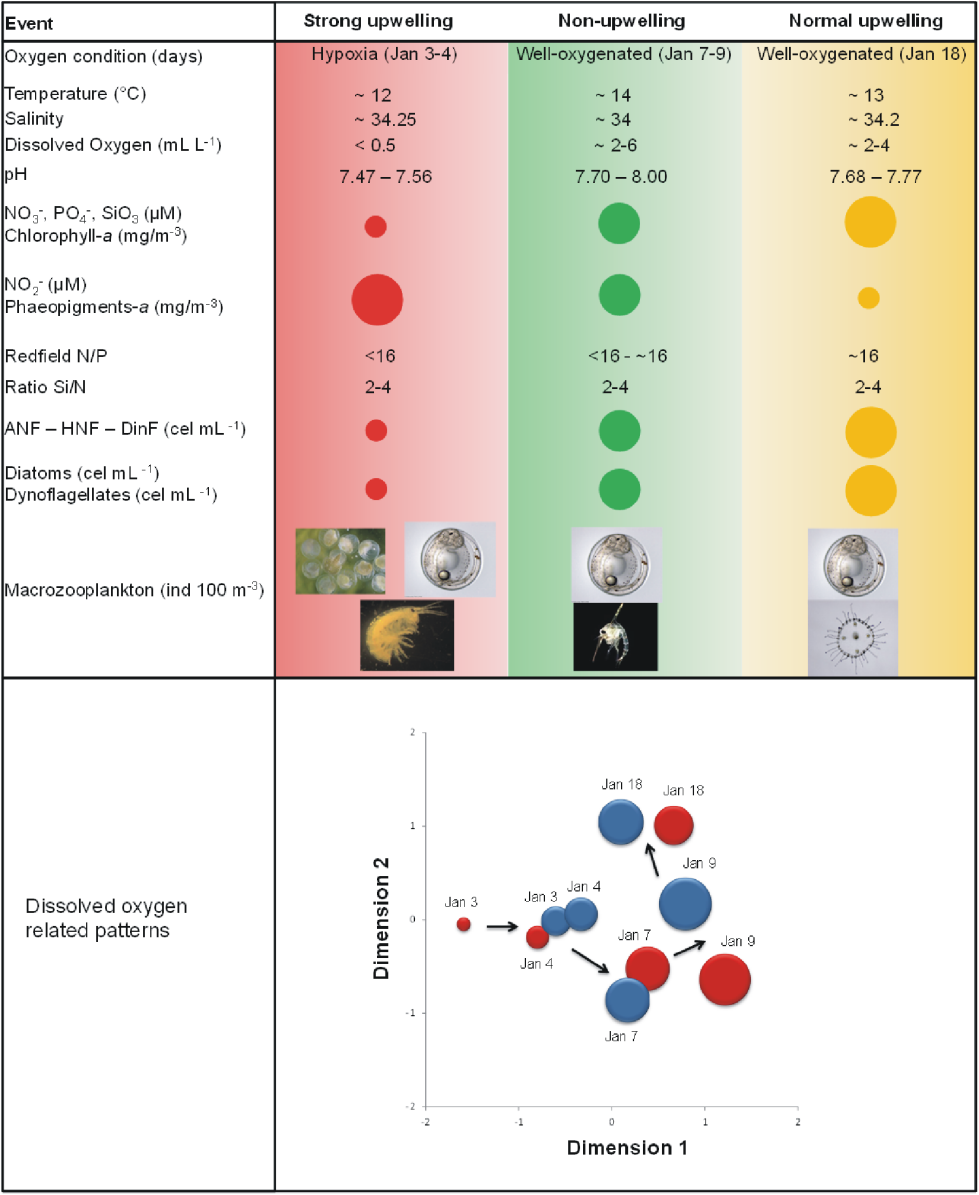
Temporal dynamics of physical-chemical and biological variables measured in Coliumo Bay during the natural hypoxia event and succeeding days. Larger diameters represent higher values for the variables quantified using circumferences. The bottom panel shows multidimensional scaling (MDS) analysis of environmental variables within and outside Coliumo Bay (red and blue circles, respectively) from January 3^rd^, to 18^th^, 2008. This analysis used Euclidian distances and normalized data (Dissolved oxygen, pH, redox, temperature, salinity, nitrate, nitrite, phosphate, silicic acid, chlorophyll *a* and phaeopigments). Circles represent the estimated centroid for each sampling day and their diameters show a proportional value of dissolved oxygen (mL O_2_ L^-1^) at the surface of the water column. Arrows show temporal multivariate environmental trends. Stress = 0.1.

**Fig 4:**
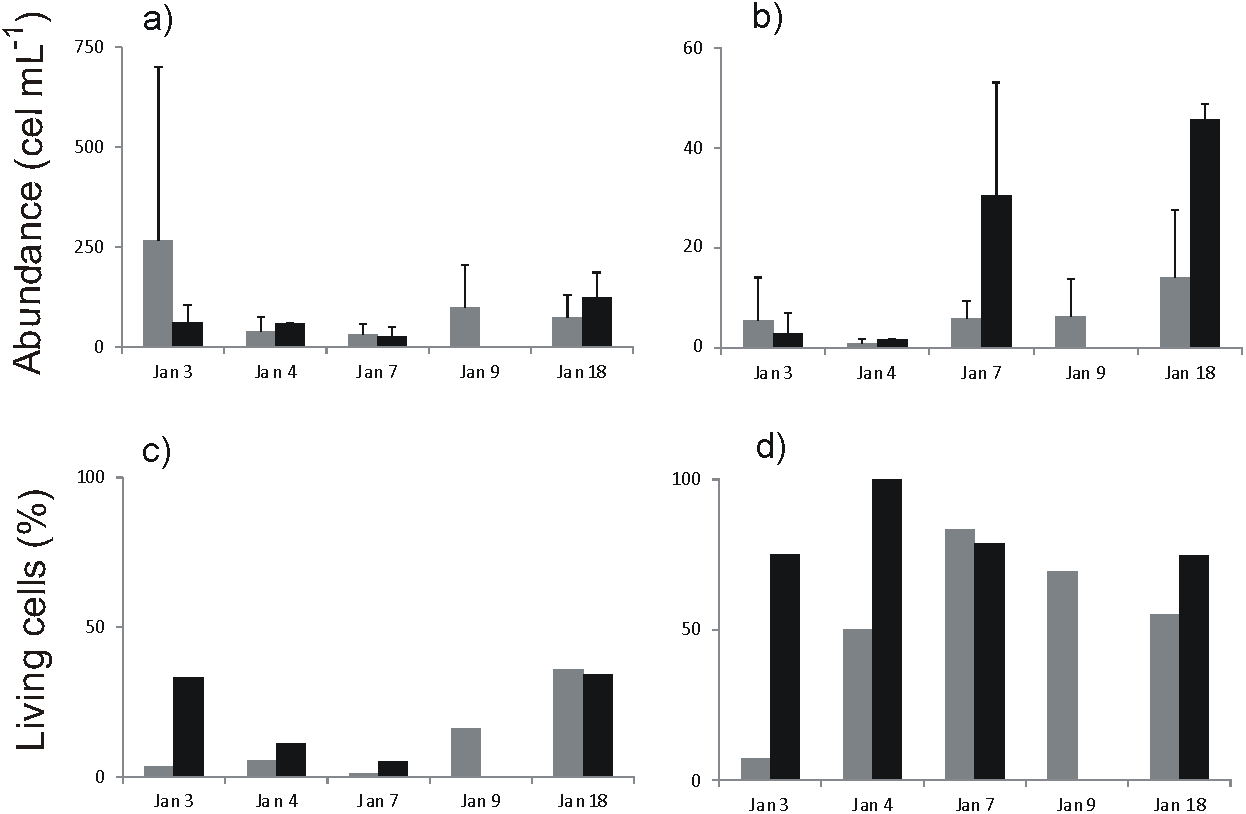
Microphytoplankton abundances. An average of (±SD) total abundance, (dead + living cells; in cells mL^-1^) of microphytoplankton in Coliumo Bay from January 3^rd^, to 18^th^, 2008. (a) Diatoms (b) dinoflagellates. (c and d) correspond to relative abundance (%) of living diatoms and dinoflagellates, respectively. The grey bars correspond to within Coliumo bay (E2, E3, E6), and the black bars correspond to outside the bay (E7 and E4).

**Fig 5:**
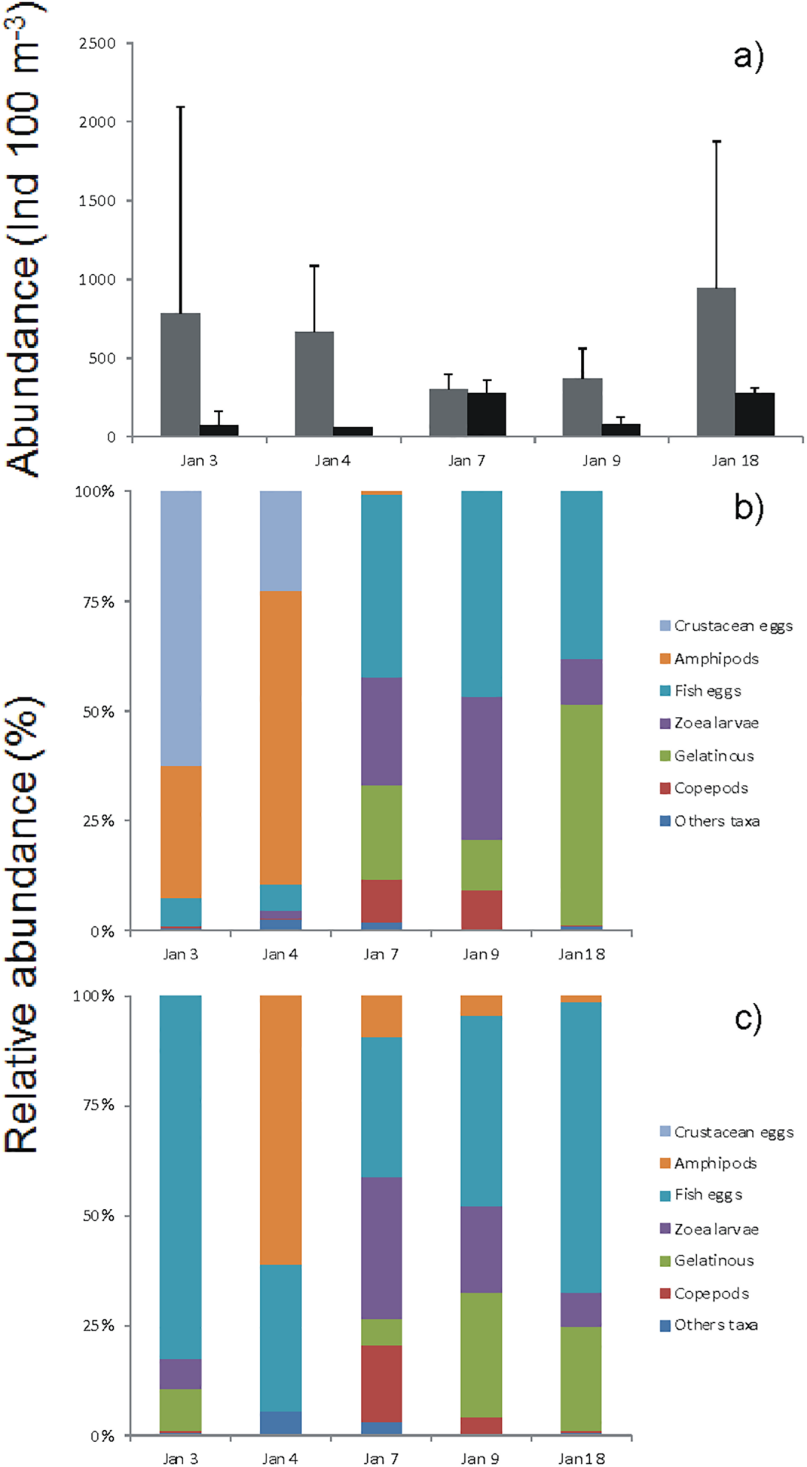
Macrozooplankton abundance during hypoxia. (a) Total macrozooplankton average (±SD) abundance (ind 100 m^-3^) in Coliumo Bay, from January 3^rd^, to 18^th^, 2008. The sampling was performed using a conical net (Mouth diameter: 50 cm). The grey bars correspond to inside Coliumo Bay (E2, E3, E6), and the black bars correspond to outside the bay (E7 and E4). (b) corresponds to relative abundance (%) by main groups in the bay (E2, E3, E6) for each sampling day and, (c) relative abundance by main groups outside the bay (E7 and E4).

**Fig 6:**
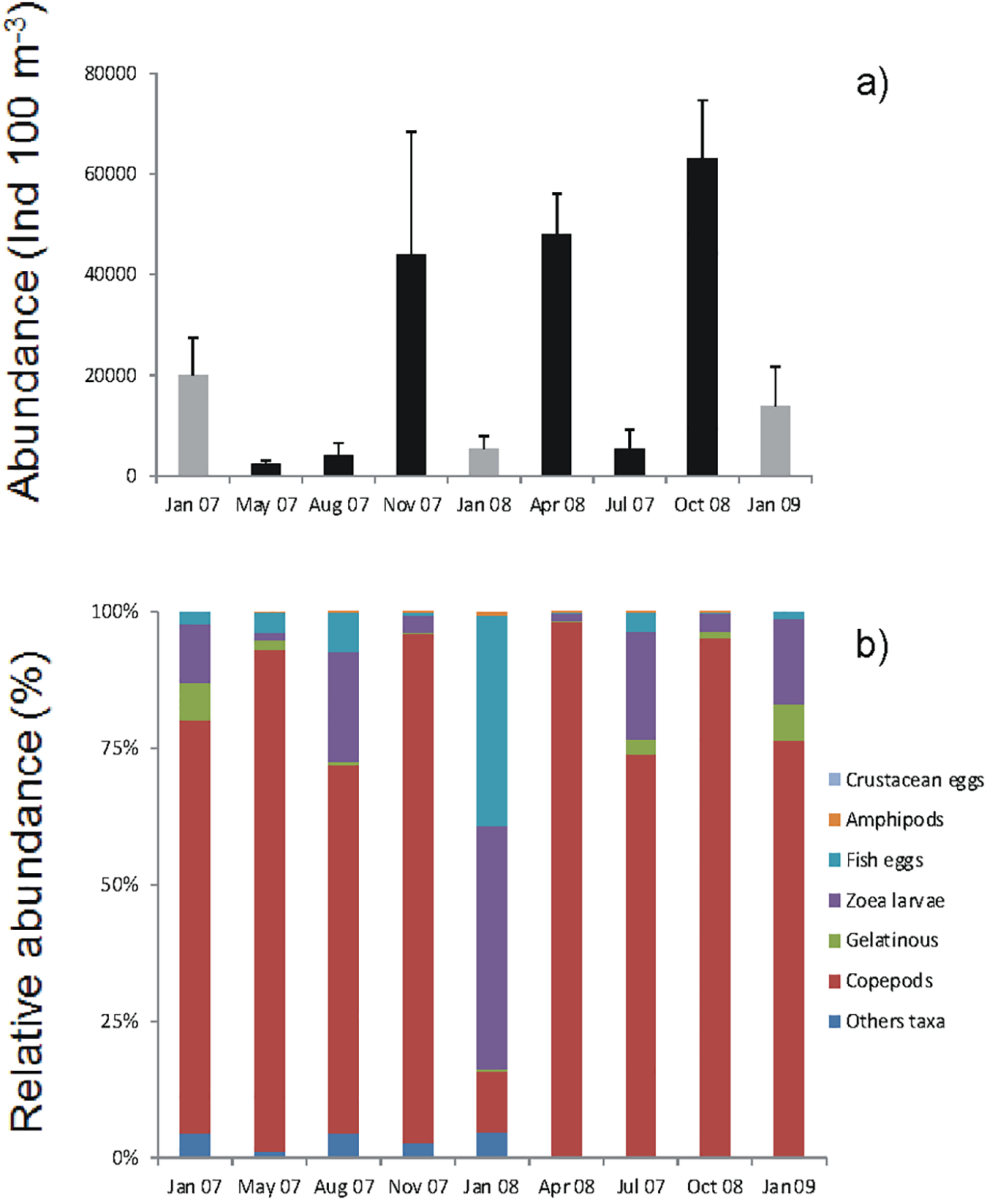
Inter-annual abundance of macrozooplankton. Total mean macrozooplankton (±SD) abundance (ind 100 m^-3^) recorded during the period January 2007—January 2009 in Coliumo Bay (sites C1, C2 and C3). Zooplankton sampling was conducted using a bongo net (mouth diameter: 60 cm). (a) Total macrozooplankton abundance. The grey bars correspond to the January sampling period for each year, and the black bars correspond to the rest of the year. (b) Relative abundance (%) by main groups for the total sampling period.

The lowest concentration of nitrate, phosphate, and silicic acid were recorded during the hypoxia event. Mean nitrite concentrations were the highest inside and outside of the bay during the hypoxia event. During the event the N:P ratio inside and outside of the bay was close to 15, increasing in the following days to nearly 16. The mean chlorophyll *a* concentration showed a tendency to increase from the hypoxia event to January 9^th^. Phaeopigments showed the opposite tendency with higher concentrations during the hypoxia event (Fig 3, See also S4 Fig). Autotrophic nanoflagellates (ANF) increased both in and outside the bay over time from the hypoxia, whereas heterotrophic nanoflagellates (HNF) and nanodinoflagellates showed the lowest abundance during the hypoxia event in and outside the bay (Fig 3, See also S5 Fig). The abundance of total diatoms in and outside the bay increased with the hypoxia event (except January 3^rd^, within the bay). The total abundance of dinoflagellates (live and dead) in and outside the bay also increased beginning with the hypoxia event (Figs 3 and 4). No live ciliates or cocolithophorids were found in the entire study period. In the case of cyanobacteria, live cells were only found on January 7^th^, although they represented nearly 40% of the total of microphytoplankton. The microphytoplankton taxa with the highest percentage of total live cells were *Chaetoceros sociales*, *Gyrodinium* sp., *Protoperidinium* sp. and *Oscillatoria submembranosa* (S1 Table). During the hypoxia, the total abundance of macrozooplankton was higher in than outside the bay (Figs 3 and 5A). The highest abundances in the bay were recorded at the beginning and the end of the hypoxia event, while outside the bay mean values increased with time (Fig 5A). The macrozooplankton assemblage in the bay during the hypoxia event was dominated by crustacean and amphipod eggs, followed by fish eggs, zoea larvae, copepods and gelatinous organisms, while outside the bay the assemblage was dominated by fish eggs and amphipods. At the end of the hypoxia event amphipods decreased, while zoea larvae, copepods and gelatinous organisms increased (Fig 5B and 5C). The macrozooplankton abundances obtained from the sampling carried out seasonally in Coliumo Bay showed that total abundance in January 2008 was lower than that of the previous and subsequent seasonal sampling periods, and also lower than in January 2007 and 2009 (Fig 6A). Copepods were the dominant group during the three sampling years (Fig 6B). Only during January 2008 the proportion of copepods decreased reaching similar relative abundance to that found within the bay during the hypoxia event (Figs 5B and 6B).

### Integrated temporal patterns

The PLS analysis that related the density of nanoplankton, microphytoplankton and macrozooplankton as a function of the set of environmental variables of the water column, produced a significant model (p = 0.036), whose first two axes together explained 70% of the total variance. The first PLS component was related mainly to the nanoplankton and microphytoplankton, while the second was mainly related to the macrozooplankton (S6 Fig). The environmental variables most related negatively to the first PLS component were surface dissolved oxygen pH at the bottom and surface, nitrate and phosphate concentration, while the largest positive values were those of nitrite and phaeopigments (S6 Fig, S2 Table). The environmental variables most related to the second PLS component (macrozooplankton) were phosphate (negative) and silicic acid, bottom temperature, chlorophyll *a* and dissolved oxygen at the bottom (positive) (S6 Fig, S2 Table). There was a significant positive correlation (r_S_ = 0.608; p<0.001) between total macrozooplankton abundance and dissolved oxygen. The majority of the taxa and/or groups of macrozooplankton also showed this pattern. Only crustacean eggs, amphipods and harpacticoid copepod groups had significant negative correlations (S3 Table).

**Fig 7:**
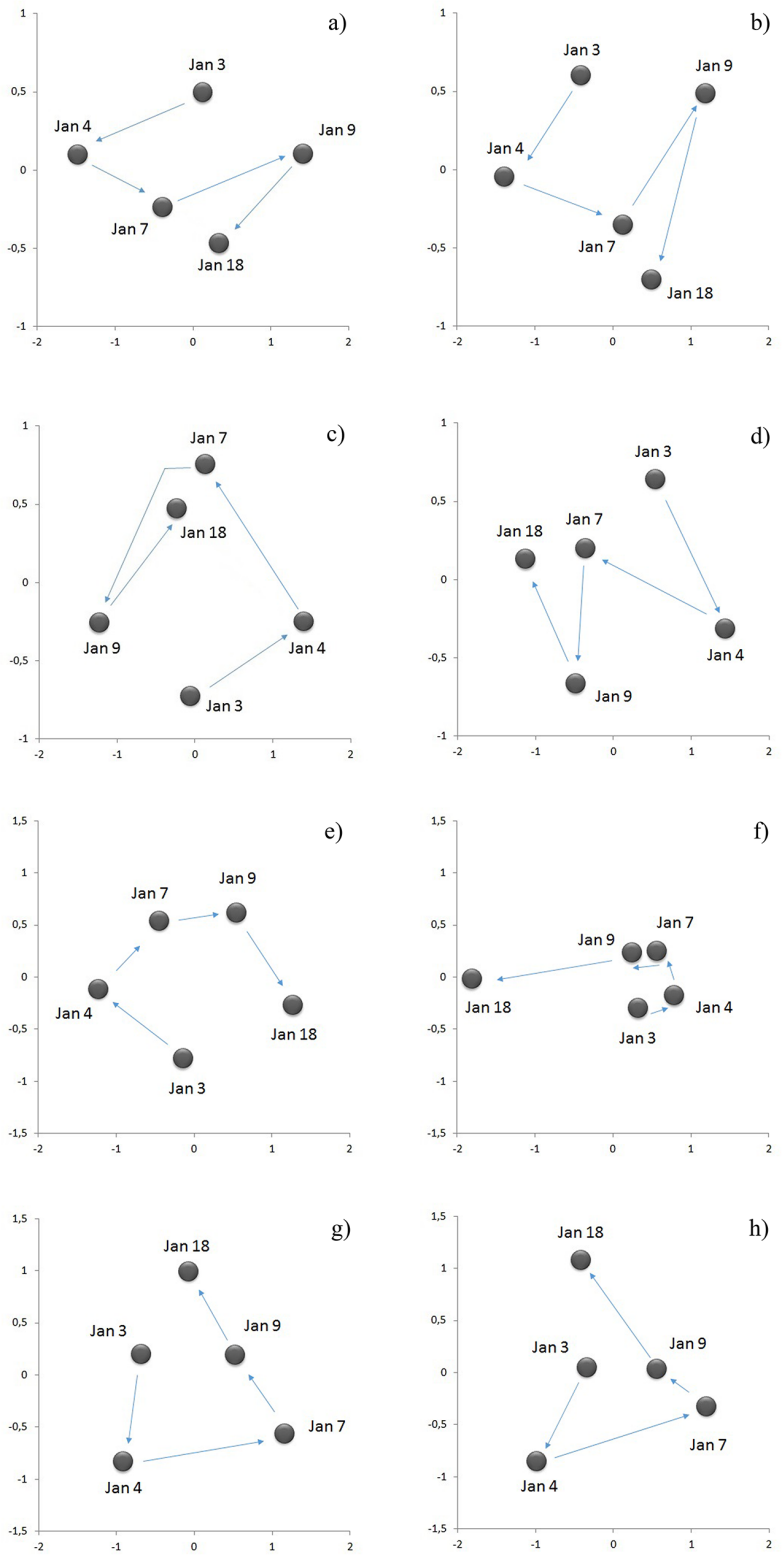
Microphytoplankton assemblage in Coliumo Bay. Non-metric multidimensional scaling (nMDS) analyses showing centroid trends of the microphytoplankton assemblage in Coliumo Bay from January 3^rd^, to 18^th^, 2008. (a) Total abundances with Bray-Curtis dissimilarity resemblance, (b) total abundances with Jaccard similarity coefficient, (c) total abundances with Bray-Curtis dissimilarity resemblance in the bay, (d) total abundances with Jaccard similarity coefficient in the bay, (e) total living cells abundances with Bray-Curtis dissimilarity resemblance, (f) total living cells abundances with Jaccard similarity coefficient, (g) total living cells abundances with Bray-Curtis dissimilarity resemblance in bay, (h) total living cells abundances with Jaccard similarity coefficient in the bay. Arrows show temporal microphytoplankton assemblage trends. In all cases stress < 0.01.

**Fig 8:**
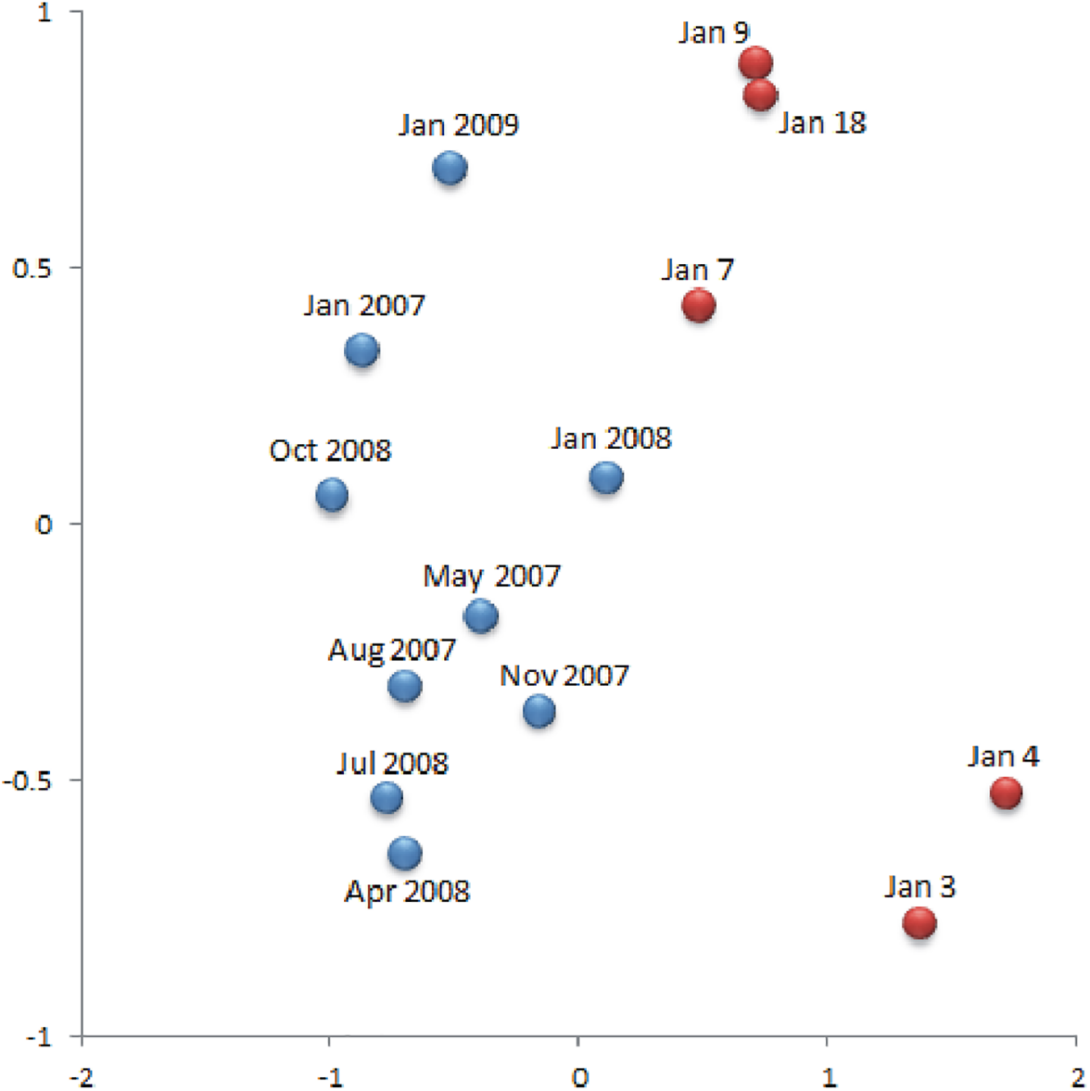
Macrozooplankton assemblage in Coliumo Bay. Non-metric multidimensional scaling (nMDS) analysis based on the presence-absence Jaccard similarity coefficient, showing temporal differences of macrozooplankton assemblage in Coliumo Bay, from the January 2007 to January 2009 seasonal sampling period (blue circles: captures with bongo net), and sampling days during the hypoxic event of January 2008 (red circles; January 3^rd^, to 18^th^, 2008, captures with conical net). Stress = 0.09.

The PERMDISP analyses, for the factors period and sampling zone, to evaluate multivariate over-dispersion of the data yielded values of p>0.01 for all the environmental variables and total and live microphytoplankton (S4 Table). Only the value *p* = 0.0004 for macrozooplankton indicates a degree of period factor over-dispersion. However, only one *pair-wise* comparison showed values p<0.01 (S4 Table), indicating that there is not an overall over-dispersion of the data and that the location results given by the PERMANOVA can be conducted. The PERMANOVA analysis, for the factor period, of the set of environmental variables of the water column showed significant differences (Table 1). The MDS analysis showed that the environmental variables have temporal dynamics and that surface dissolved oxygen (indicator of hypoxia) shows directionality beginning on January 3^rd^, i.e. with the onset of the hypoxia event (Fig 3). A similar temporal tendency was shown by the nMDS analyses performed with the mean abundances of total and live microphytoplankton cells (Fig 7). The PERMANOVA analyses, for the period factor, performed for total and live microphytoplankton also showed significant differences, both for species relative abundances and species presence-absence (Tables 2 and 3). The macrozooplankton nMDS analysis based on the Jaccard coefficient for presence-absence of species, integrating both seasonal sampling and the sampling conducted during the hypoxia event, shows that during the hypoxia (January 3^rd^, and 4^th^, 2008,) the assemblage was less similar than the other sampling periods (Fig 8). The PERMANOVA analyses for macrozooplankton showed significant differences among periods (Table 4). The *pair-wise* tests show differences among the three periods (S5 Table). Finally, the samples of January 7^th^, 9^th^, and 18^th^, 2008, were more similar to the inter-annual seasonal sampling of January 2007, 2008 and 2009 (Fig 8).

**Table 1:** PERMANOVA output for the environmental variables of the water column.

Source	Df	SS	MS	Pseudo-*F*	*P*(perm)	Unique perms
**Period**	1	60.623	60.623	5.7954	**0.0009**	9945
**Zone**	1	11.554	11.554	1.1046	0.3287	9936
**P x Z**	1	6.3601	6.3601	0.60801	0.6774	9931
**Res**	19	198.75	10.461			
**Total**	22	308				

The “period” factor represents the sampling day (January 3^rd^, 4^th^, during hypoxia, 7^th^, 9^th^, and 18^th^, after hypoxia). The “zone” factor refers to sampling sites located inside or outside Coliumo Bay. Resemblance analysis was conducted with Euclidian distances and normalized data. In bold, *p* values < 0.05.

**Table 2:** PERMANOVA output for total microphytoplankton cells.

**(a) Source**	**df**	**SS**	**MS**	**Pseudo-*F***	***P*(perm)**	**Unique perms**
**Period**	1	6259.4	6259.4	1.9229	**0.0111**	9900
**Zone**	1	3490.6	3490.6	1.0723	0.3654	9910
**P x Z**	1	3512.8	3512.8	1.0791	0.3581	9914
**Res**	17	55338	3255.2			
**Total**	20	69342				
**(b) Source**	**df**	**SS**	**MS**	**Pseudo-*F***	***P*(perm)**	**Unique perms**
**Period**	1	5813.1	5813.1	2.1723	**0.0024**	9902
**Zone**	1	2430.7	2430.7	0.90833	0.5859	9909
**P x Z**	1	2614.2	2614.2	0.97692	0.4920	9896
**Res**	17	45492	2676			
**Total**	20	57244				

(a) Bray Curtis non-transformed data: compositional density of total cells

(b) Jaccard: species presence-absence of total cells. The “period” factor corresponds to sampling days (January 3^rd^ and 4^th^, during hypoxia, 7^th^, 9^th^, and 18^th^, after hypoxia). The factor “zone” refers to sampling sites located inside or outside Coliumo Bay. In bold *p* values < 0.05.

**Table 3:** PERMANOVA output for live microphytoplankton cells.

**(a) Source**	**df**	**SS**	**MS**	**Pseudo-*F***	***P*(perm)**	**Unique perms**
**Period**	1	7591.7	7591.7	1.964	**0.0129**	9900
**Zone**	1	3952.8	3952.8	1.0226	0.4201	9920
**P x Z**	1	6584.7	6584.7	1.7035	**0.0424**	9925
**Res**	17	65712	3865.4			
**Total**	20	84965				
**(b) Source**	**df**	**SS**	**MS**	**Pseudo-*F***	***P*(perm)**	**Unique perms**
**Period**	1	6429.2	6429.2	1.747	**0.0339**	9903
**Zone**	1	2921.5	2921.5	0.79385	0.6973	9907
**P x Z**	1	6365.2	6365.2	1.7296	**0.0358**	9917
**Res**	17	62563	3680.2			
**Total**	20	78254				

(a) Bray Curtis non-transformed data: compositional density

(b) Jaccard: species presence-absence. The factor “period” corresponds to sampling day (January 3^rd^, and 4^th^, during hypoxia, 7^th^, 9^th^, and 18^th^, after hypoxia). The factor “zone” refers to sampling sites located inside or outside Coliumo Bay. In bold, *p* values < 0.05.

**Table 4:** PERMANOVA output for macrozooplankton in Coliumo Bay from January 2007 to January 2009, including January 3^rd^,-18^th^, 2008.

Source	df	SS	MS	Pseudo-*F*	*P*(perm)	Unique perms
**Period**	2	35949	17974	9.7641	**0.0001**	9902
**Res**	74	1.3622E5	1840.9			
**Total**	76	1.7217E5				

The factor “period” corresponds to sampling days (before, during, and after hypoxia). The resemblance analysis was obtained for species presence-absence data with Jaccard coefficient. In bold *p* values < 0.05.

## Discussion

### Upwelling processes and functionality of the coastal pelagic ecosystem

This study details the changes that occurred in the pelagic coastal ecosystem of Coliumo Bay as a resultof the intrusion of hypoxic water (ESSW) from the OMZ during an intensive upwelling event in the summer of 2008. The effects of this natural perturbation, observed in a time window of days, suggest alterations in physical, chemical and biological components of the ecosystem: hydrography, nutrient concentrations and nutrient ratios, photosynthetic pigments and abundances of nanoplankton, microphytoplankton and macrozooplankton (Fig 3). Three dynamic states of the ecosystem can be identified during this process. First, the beginning of the upwelling event on January 3^rd^ and 4^th^ showed a hypoxic water column, low temperatures and high salinities. Redox, pH, nitrate, phosphate, silicate and chlorophyll-*a* levels were at their lowest of the entire study period, while nitrite and phaeopigments were at their highest. At this stage, the Redfield ratio was N:P < 16, the abundances of nanoplankton and microphytoplankton were the lowest and the latter showed the smallest proportion of live organisms. Macrozooplankton abundance was at its highest, dominated mainly by crustacean, fish eggs and amphipods. The second and third stages (January 7^th^,to 9^th^, and, 18^th^), which correspond to the relaxation period and the least intense upwelling period, showed a continuum process with tendencies that were the reverse of the initial state for the majority of the abiotic and biotic variables. Dissolved oxygen indicated a well oxygenated water column, with no major changes in temperature or salinity. Redox, pH, nitrate, phosphate, silicate and chlorophyll-*a* concentrations increased, while those of nitrite and phaeopigments decreased. The Redfield ratio stabilized near its theoretical value of 16 [49], and nanoplankton and microphytoplankton increased in abundance, the latter with a greater proportion of live organisms. The total abundance of macrozooplankton tended to decrease from (January 7^th^ to 9^th^), and then increased towards the end of the sampling period, with a change in taxonomic composition that was dominated by fish eggs and gelatinous organisms.

It is known that changes in nutrient concentrations and ratios strongly influence the composition and abundance of phytoplankton communities [50]. Deviations from the Redfield ratio implies that the nutrient with less availability is likely to become limiting for phytoplankton growth [16, 51]. During the initial stage of the hypoxia event we found a decrease in the Redfield ratio (N:P < 16), which normalized on January 7^th^, and 8^th^ (relaxation period). This would be explained by a decrease in and/or alteration of the concentrations of nitrate and phosphate at the beginning of the hypoxia event as a product of a water column with reduced conditions (i.e. negative redox potential between -3 and -31 on January 3^rd^), when the highest values of nitrite were also recorded (Fig 3). When more oxidant conditions returned to the water column in the following days, nitrate and phosphate concentrations increased. Higher concentrations of nitrite than nitrate have been described for coastal ecosystems of the HCS influenced by ESSW poor in dissolved oxygen [52, 53], as was observed during this acute hypoxia event. As well, throughout the period sampled the Si:N ratio was greater than 1 and without major temporal changes. This would theoretically produce a dominance of diatoms in the microphytoplankton [54, 55], which we observed mainly within the bay.

Variations in the stoichiometric proportions of nutrients may also be functionally associated with changes in the abundances of different phytoplankton groups. For example, after the days of hypoxia we observed growing abundance of autotrophic nanoflagellates and nanodinoflagellates, an indication that nanophytoplankton, which belong to the smallest size class, recovered abundance rapidly, only days after the hypoxia event. This pattern was also observed earlier for heterotrophic nanoflagellates, perhaps associated with greater availability of dead microphytoplankton cells. Not only was the lower abundance of larger phytoplankton during the days of hypoxia notable, but also the high proportion of dead and/or inactive cells of the microphytoplankton, which in many cases was 100%. The high mortality is concordant with the greater concentration of phaeopigments recorded for the days of acute hypoxia (Fig 3). The relation between cell abundance and phaeopigment concentration appears to be very close during the entire study period. Thus, after the period of hypoxia the proportion of dead microphytoplankton cells decreased, their abundance increased and the concentration of phaeopigments decreased, all related to a re-oxygenated water column (Fig 3). Although this relation may be explained by the mortality of microphytoplankton in the bay due to the hypoxia, part of it may have been due to a mechanism of transportation of dead cells to the coastal zone by ESSW with low dissolved oxygen from the deeper zone of the continental shelf. In this scenario of possible combined effects, since the microphytoplankton composition in this study was not different from those reported previously for Coliumo Bay by [56], we hypothesize that the intrusion of ESSW, which has a low oxygen content, was the most likely cause for the mortality of the resident microphytoplankton, rather than importation of dead cells from the continental shelf by advective transport. For instance, Stauffer et al [57] stated that during an extreme hypoxia that took place in California significant changes in phytoplankton community structure were observed together with dramatically reduced photosynthetic yield of the remaining species, indicating severe physiological stress. In addition, the strong temporal sequence of dissimilarity observed in the nMDS for the phytoplankton inside and outside the bay (Fig 7) suggests that the temporal dynamics of taxa exchange (observed both for relative abundances and presence-absence) was associated with rapid population growth of the surviving species. It should be noted that the temporal sequences observed in the different nMDS estimated for the microphytoplankton were also observed for the set of all the hydrographic variables and nutrients in the water column during the hypoxia event (Fig 3), suggesting a close functional relation between environmental conditions and microphytoplankton cells.

To analyze the consequences on macrozooplankton, the temporal dynamics of two variables, abundance and composition, must be considered. We observed an apparent paradox in abundance in this hypoxia event that is the greatest abundance of macrozooplankton was recorded on January 3^rd^, and 4^th^, days of acute hypoxia. However, the abundances were represented mainly by crustacean eggs and amphipods. The former are probably associated with the mortality of adult organisms (e.g. the crab *Cancer coronatus*), which occurred in the bay during the hypoxia [19], while the latter was due to vertical migration and/or free-floating death individuals, increasing their abundance in the shallowest layer of the water column. Neither dominant taxa in the water column during the hypoxia are characteristic representatives of the plankton community in Coliumo Bay [58]. Although in lower abundance, fish eggs were also dominant outside the bay during the days of hypoxia. These may also be associated with the massive mortality of adult fish during the hypoxia event within the bay [18, 19]. In the days after the hypoxia, the macrozooplankton ensemble within the bay was dominated by fish eggs and gelatinous organisms. Towards the end of the study period, after the relaxation and new upwelling event, total abundances were greater, suggesting a numerical recovery of the components of the community given by the presence of zoea states of different crustacean taxa. In the hypoxia month of January 2008 we observed a notable decrease in macrozooplankton total abundance and in all dominant zooplankton taxa (e.g. copepods) compared to the trimestral samples of January 2007 (before) and 2009 (after) the hypoxia event. As in the case of microphytoplankton, the massive mortality of macrozooplankton due to the hypoxia may also be due to the advective transport of organisms to and from the bay or to their evasion of water low in dissolved oxygen (*e*.*g*. [59, 60]). The change in the composition of the ensemble present in January 2008 and the positive correlation of total macrozooplankton and the majority of the groups with the concentration of dissolved oxygen (Figs 5 and 6; see also S3 Table) suggests that the most plausible cause of low abundances and species replacement was the abrupt decrease in the concentration of dissolved oxygen and changes in the hydrographic-environmental conditions of the water column, resulting in lower abundances for the entire hypoxia event, as well as effects on total abundance at an inter-annual time scale.

### Response of organisms to hypoxia

The organisms that inhabit the marine coastal zone of the HCS are periodically exposed to low concentrations of dissolved oxygen produced by upwelling events. Thus, their ecological and evolutionary success has been the result of developing adaptive mechanisms. For example, many species that inhabit upwelling areas have developed metabolic adaptations to cope with hypoxia (*e*.*g*. [4, 61]) that have allowed them to survive hypoxic events that can last for days. Some components of the macrozooplankton community that are less adapted to low oxygen concentrations have developed biological mechanisms like evasive behavior, limiting them to live in the surface ocean layer [61–63] or maintain a spatial distribution near the coast where dissolved oxygen concentrations are greater [64]. Nevertheless, many species do not have adaptive and/or behavioral mechanisms to cope with hypoxic conditions. Consequently, when sudden natural hypoxia events occur due to coastal upwelling, mass mortality and large-scale beaching of organisms can occur [5, 18]. Mortality is taxonomic-dependent, with fish and crustaceans being the most affected groups [12]. During this natural hypoxia event, as well as observing the beaching of fish and juvenile and adult crustaceans in the intertidal zone of the bay and the surrounding area [18, 19], we recorded the beaching of macrozooplankton organisms, especially mysidaceans of the genus *Neomysis* sp., which reached densities of *ca*. 10,000 ind. * 100 cm^-2^ (See Fig 1). It is possible that these organisms, due to the sudden and extensive event within the bay could not avoid hypoxic water, even though day-night vertical migration behavior has been described [65]. Vertical migrations and high abundances of *Neomysis* sp. in the water column, mainly at night, have been described in Coliumo Bay [66, 67]. Their abundance reaches up to *ca*. 1,500 ind. * 10 m^-3^ in the superficial stratum of the bay at night, and one or two orders lower in the surface and the bottom during the day [66, 67]. The absence of these organisms in the water column during the hypoxia event (only 0.68 ind. * 10 m^-3^ on January 3^rd^, in the E6 site suggests that the acute effect of the hypoxia was dramatic for the population. However, this acute effect apparently has a threshold (i.e. < 1 mL O_2_ L^-1^) rather than a gradual response. This is based on the non-significant correlation between the concentration of dissolved oxygen and the abundance of *Neomysis* sp. estimated for all samples from January 2007 and January 2009 (see S3 Table); all of them under well oxygenated environmental conditions.

The effects of acute hypoxia were also detectable at an inter-annual scale. For example, the copepod *Acartia tonsa*, which is the most abundant and dominant of the macrozooplankton ensemble in the bay, was completely absent during the hypoxia event (January 3^rd^, and 4^th^), with a slight recovery in the following days. It has been described that although this copepod is capable of surviving dissolved oxygen concentrations as low as 1 mL O_2_ L^-1^, below this threshold it shows high mortality [17, 68–70]. This concurs with the strong positive correlation estimated between the density of *A*. *tonsa* and dissolved oxygen between January 2007 and January 2009 (r = 0.747, p < 0.001. This pattern was also reflected in other components of the ensemble, mainly copepods, with low abundance or total absence at dissolved oxygen concentrations below 1 mL O_2_ L^-1^ in the January 2007-January 2009 period. Other relationships between copepods abundance and dissolved oxygen concentrations have been described for the northern zone of the HCS with contrasting results [62]. Negative relationships have been found between community parameters of the copepod assemblage and the depth of the OMZ for the study zone, however, relations have not been reported with respect to the concentration of dissolved oxygen [71, 72]. Stalder and Marcus [70] experimented with the copepods *A*. *tonsa*, *Labidocera festiva* and *Centropages hamatus*, obtained from a geographic area where hypoxia events do not usually occur. The organisms were placed in tanks where hypoxia events in the bottom layer were simulated; however, none of the three species showed evasive behavior, suggesting that in coastal zones where hypoxia develops suddenly, the decrease in copepod abundance is mainly due to mortality rather than evasive behavior and/or advective transport. Decker et al. [73] found geographic differences in the hypoxia evasion behavior of two populations of *A*. *tonsa*, one in the Chesapeake Bay estuary (USA) and the other in Florida (USA). The former, which has historically been exposed to gradients of dissolved oxygen in the water column, evaded areas with low concentration, while the latter, which is not usually exposed to low dissolved oxygen, did not show evasive behavior from lethal levels of dissolved oxygen and was thus more vulnerable to hypoxia events. Since the coast of south-central Chile has had upwelling and associated hypoxia events since at least the last post-glacial period [74], it is probable that copepod species and other taxa of macrozooplankton resident in the HCS have developed evolutionary strategies of evasive behavior that have allowed them to minimize their mortality due to low dissolved oxygen concentrations in spring and summer. Further, not only acute lethal effects related to hypoxia are important for copepods; sub-lethal effects may also have consequences over a longer time scale. It has been reported that concentrations below 1 mL O_2_ L^-1^ inhibit egg eclosion of *A*. *tonsa* [17] and decrease egg production [68, 69], even if food is abundant [75]. Copepods also tend to develop more slowly and mature at smaller sizes than in well oxygenated environmental conditions [69]. The above suggests that events of acute hypoxia in shallow coastal zones may also decrease copepod abundance, especially *A*. *tonsa*, even with a time lag. This may have strong repercussions on population dynamics. For example a reproductive failure may cause a cascade effect on coastal trophic webs, especially for species that feed on these micro-crustaceans (*e*.*g*. sardines and anchovies).

There is information on the composition and abundance of the microphytoplankton community for this study zone, as well as its temporal variability with respect to oceanographic conditions [56, 76–79]. However, these studies did not evaluate the quantitative effect of acute natural hypoxia on survival. The proportions of live/dead cells for the different groups recorded in this study are the first reported, which in the case of the microphytoplankton mortality was very high. A similar pattern of temporal change in the microphytoplankton community, in the concentration of chlorophyll-*a*, and nutrients was recorded for an acute hypoxia event in the northern basin of King Harbor in California [57]. According to Stauffer et al. [6] the acute hypoxia was due to the consumption of dissolved oxygen by respiration due to an exceptionally high density of the clupeiform fish *Sardinops sagax*. These authors also indicated a strong decline in the photosynthetic yield of the phytoplankton that survived the hypoxia due to severe physiological stress.

Recently Gobler and Baumann [80] reviewed the evidence from published factorial experiments that combine pH and DO levels on different traits, life stages and species across a broad taxonomic spectrum. They found that the most common response was additive negative effects of combined low pH and low dissolved oxygen although they also reported synergistic negative effects. It is well known that OMZ waters have low pH and low oxygen content in comparison to the ocean surface waters [80–82]. At the beginning of the hypoxia event at Coliumo Bay pH and dissolved oxygen were at the lowest levels suggesting a possible negative synergic effect on the survival of phytoplankton and zooplankton.

Future studies should also be oriented to determining how much of the decreased abundance of microphytoplankton and macrozooplankton during acute hypoxia events is due to advective transport out of the shallow coastal zone. For example, Escribano and Hidalgo [62] described advective transport in response to upwelling as a mechanism of zooplankton loss in a deeper coastal zone. The authors proposed a two-layer mechanism, where surface water is transported offshore exporting individuals to the more oceanic zone, while a deep layer transports them back to the coastal zone. However, this mechanism of loss by transport may be attenuated by the vertical migration of individuals [83], which in any case would be limited by the presence of water low in dissolved oxygen [17]. The net balance of mortality and loss by transport, both of microphytoplankton and macrozooplankton, is still to be resolved for this and other shallow coastal bays. Finally, since coastal upwelling generally occurs at scales of over tens of kilometers, it is probable that what we documented in this shallow bay has occurred in other shallow, protected zones of the HCS. In fact, according to satellite images obtained for this acute hypoxia event, these conditions were repeated in other zones over an area of nearly 500 linear km of the coast (see S1 Fig). In the future it would be relevant to evaluate the synergic effects that global warming will have on the coastal ecosystems of the HCS. It has been suggested that upwelling events produced by southwest winds will increase due to global warming, thus favoring the intensity and magnitude of natural hypoxias [84]. If this prediction is correct, our results suggest that the effect of natural hypoxia produced by upwelling in the coastal zone of the HCS may be dramatic and have important consequences for pelagic and benthic species that are not adapted to overcoming environments with low concentration of dissolved oxygen.

## Supporting information

S1 Fig Satellite images of the study zone from December 30^th^, 2007 to January 31^st^, 2008. Images were obtained from the ANTARES Observation Network (http://antares.ws) and show the temporal dynamics of the upwelling during the hypoxia event. White arrows indicate the location of Coliumo Bay.
(DOCX)

S2 Fig Time series of the wind intensity/direction, sub-tidal temperatures, and tide height for the study before, during, and after the hypoxia event. The time series of the intensity/direction (U and V vectors) of the winds (a), sub-tidal temperature (b) and tide height (c) for the study before, during and after the hypoxia event. The dashed line indicates the day of massive mortality and the beaching of organisms (January 3^rd^). The grey polygon indicates the time period in which the hydrographic, physical-chemical and biological samples were obtained.
(DOCX)

S3 Fig Vertical sections of dissolved oxygen, temperature and salinity for Coliumo Bay during the hypoxia in January 2008. Vertical sections of: (a, b) dissolved oxygen (mL O_2_ L^-1^), (c, d) temperature (°C) and (e, f) salinity for Coliumo Bay during the hypoxia in January 2008. Dots indicate the depths at which the hydrographic data were obtained and used for the contour fitting with Kriging interpolation. The panels on the left are the stations located outside the bay (E7 and E4); those on the right are the stations inside the bay (E2, E3 and E6).
(DOCX)

S4 Fig Nutrients and pigments concentrations. Average (±SD) concentration (in μM) of (a) nitrate, (b) nitrite, (c) phosphate and, (d) silicic acid. Also shown are average ratios of (e) N:P and, f) Si:N. Plots (g and h) correspond to average concentrations of chlorophyll *a* (mg m^-3^) and phaeopigments (mg m^-3^), respectively. All measurements are the integrated values of the entire water column in Coliumo Bay from January 3^rd^, to 18^th^, 2008. The grey bars correspond to inside Coliumo Bay (E2, E3, E6), and the black bars correspond to outside the bay (E7 and E4). There was no pigment data for January 18^th^.
(DOCX)

S5 Fig Nanoplankton and dinoflagellates abundances. Average (±SD) abundance (cells mL^-1^) of total nanoplankton in Coliumo Bay from January 3^rd^, to 18^th^, 2008 (a) autotrophic nanoflagellates (ANF), (b) heterotrophic nanoflagellates (HNF), (c) dinoflagellates. The grey bars correspond to inside Coliumo Bay (E2, E3, E6), and the black bars correspond to outside the bay (E7 and E4).
(DOCX)

S6 Fig Partial least squares (PLS) analysis for environmental variables during the hypoxic event (January 3^rd^, to 18^th^, 2008). The first and second component explained 37% and 33% of the total variance, respectively. Environmental variables are surface (-S) and bottom (-b) temperature (T), salinity (Sal), dissolved oxygen (Ox), pH, and redox (R). Average water column nutrients (Nitrate, nitrite, phosphate, silicic acid) and pigments (Chlorophyll *a*, phaeopigments) were also used in the analysis.
(DOCX)

S1 Table Summary of the microphytoplankton taxa in Coliumo Bay. (DOCX)

S2 Table Results of PLS regression for macrozooplankton, microphytoplankton, and nanoplankton density as a function of environmental variables. (DOCX)

S3 Table Spearman correlations between the most abundant macrozooplankton taxa and concentration of surface dissolved oxygen. (DOCX)

S4 Table (a) PERMDISP output for the analyses conducted for environmental and biological variables in Coliumo Bay. (b) PERMDISP *pair-wise* output for macrozooplankton inside Coliumo Bay.
(DOCX)

S5 Table PERMANOVA *pair-wise* output for macrozooplankton inside Coliumo Bay. (DOCX)

S1 Database Macrozooplankton and microphytoplankton composition and abundance data. (XLSX)
