# Supplementary figures and images for: Short-term alteration of biotic and abiotic components of the pelagic system in a shallow bay produced by a strong natural hypoxia event
Source: PLoS One. 2017 Jul 17;12(7):e0179023. doi: 10.1371/journal.pone.0179023 (PMC5513412; doi:10.1371/journal.pone.0179023)

**Supporting Information (S1 Fig)**

**
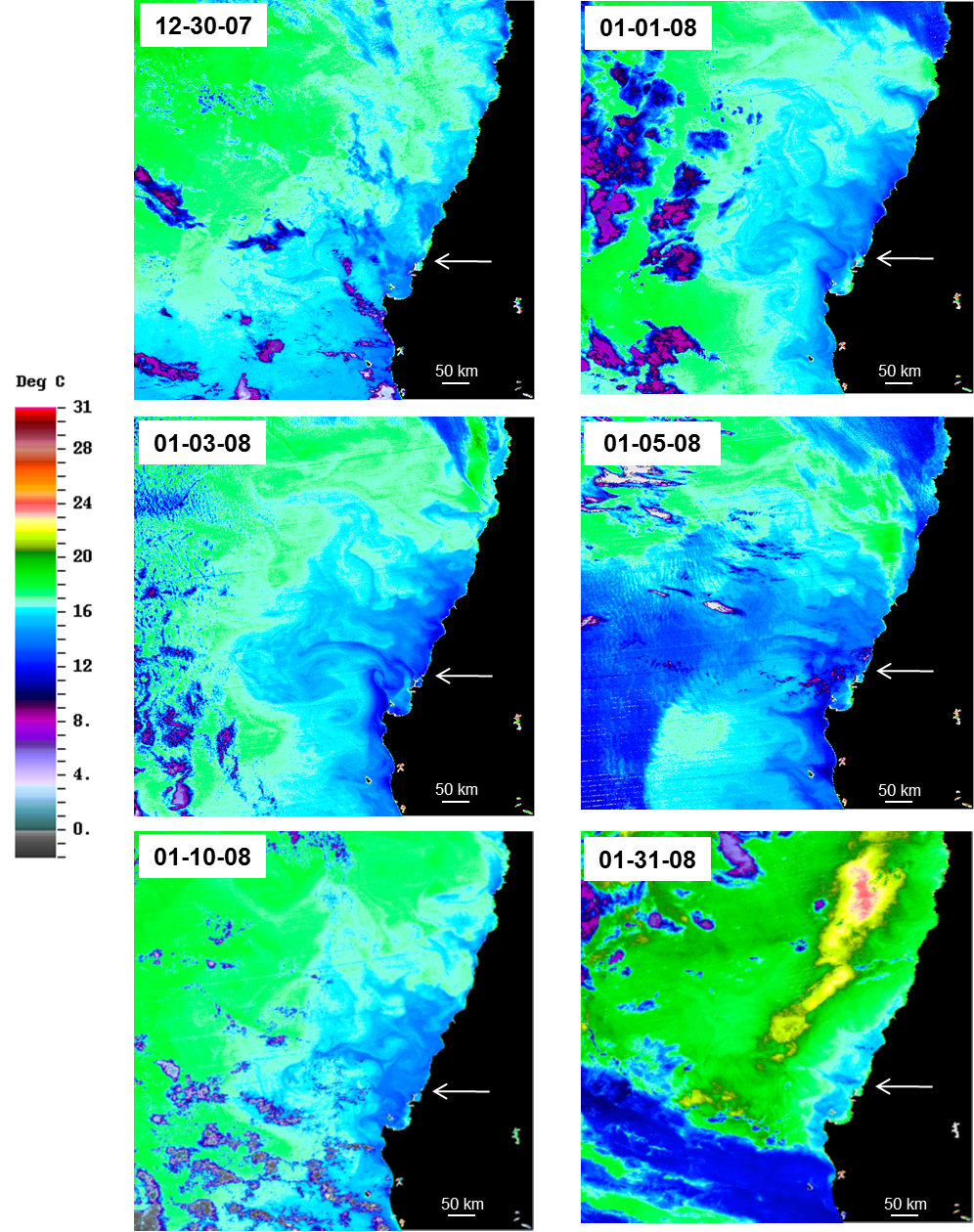
**

Supplement: S1 Fig — Images were obtained from the ANTARES Observation Network (http://antares.ws) and show the temporal dynamics of the upwelling during the hypoxia event. White arrows indicate the location of Coliumo Bay. (DOCX) [file pone.0179023.s001.docx]

**Supporting Information (S3 Fig)**

**
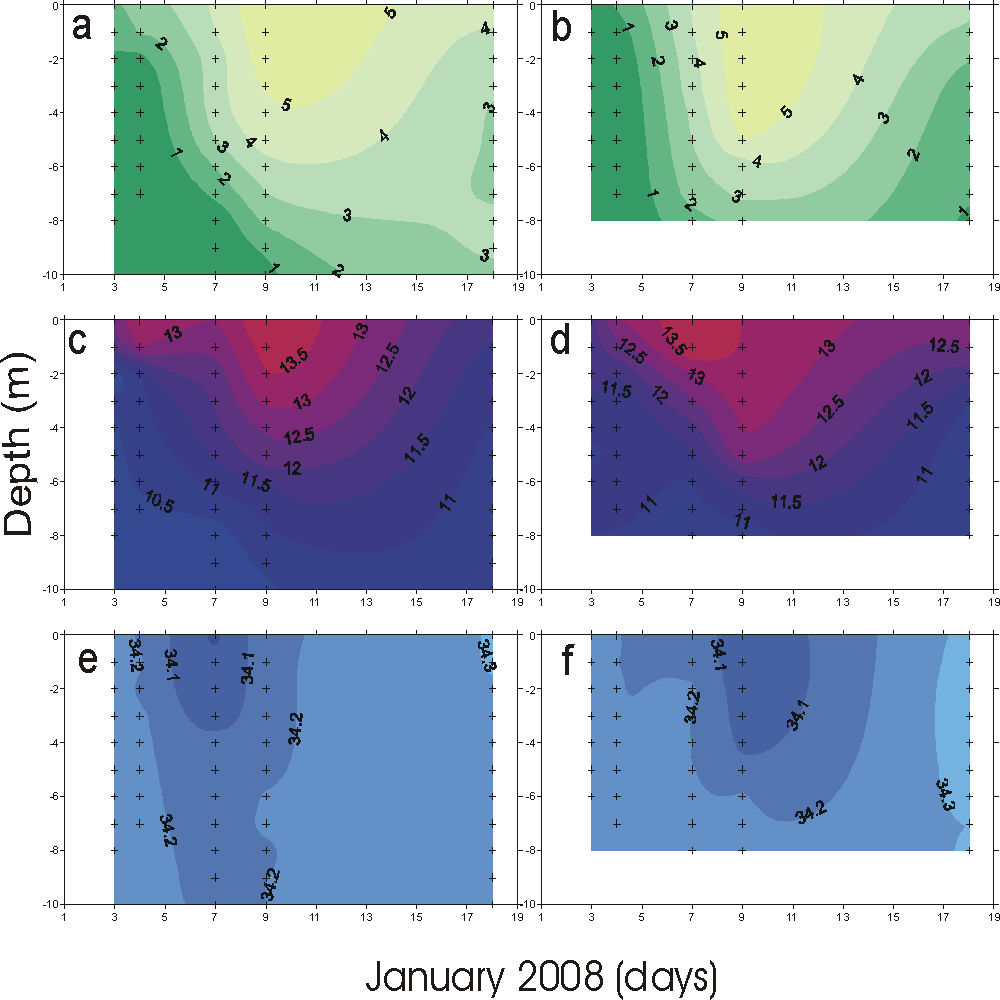
**

Supplement: S3 Fig — Vertical sections of: (a, b) dissolved oxygen (mL O2 L-1), (c, d) temperature (°C) and (e, f) salinity for Coliumo Bay during the hypoxia in January 2008. Dots indicate the depths at which the hydrographic data were obtained and used for the contour fitting with Kriging interpolation. The panels on the left are the stations located outside the bay (E7 and E4); those on the right are the stations inside the bay (E2, E3 and E6). (DOCX) [file pone.0179023.s003.docx]
