# Supplementary material for: Short-term alteration of biotic and abiotic components of the pelagic system in a shallow bay produced by a strong natural hypoxia event
Source: PLoS One. 2017 Jul 17;12(7):e0179023. doi: 10.1371/journal.pone.0179023 (PMC5513412; doi:10.1371/journal.pone.0179023)
Supplement: S2 Fig — The time series of the intensity/direction (U and V vectors) of the winds (a), sub-tidal temperature (b) and tide height (c) for the study before, during and after the hypoxia event. The dashed line indicates the day of massive mortality and the beaching of organisms (January 3rd). The grey polygon indicates the time period in which the hydrographic, physical-chemical and biological samples were obtained. (DOCX) [file pone.0179023.s002.docx]

**Supporting Information (S2 Fig)**
