# Supplementary material for: Short-term alteration of biotic and abiotic components of the pelagic system in a shallow bay produced by a strong natural hypoxia event
Source: PLoS One. 2017 Jul 17;12(7):e0179023. doi: 10.1371/journal.pone.0179023 (PMC5513412; doi:10.1371/journal.pone.0179023)
Supplement: S4 Fig — Average (±SD) concentration (in μM) of (a) nitrate, (b) nitrite, (c) phosphate and, (d) silicic acid. Also shown are average ratios of (e) N:P and, f) Si:N. Plots (g and h) correspond to average concentrations of chlorophyll a (mg m-3) and phaeopigments (mg m-3), respectively. All measurements are the integrated values of the entire water column in Coliumo Bay from January 3rd, to 18th, 2008. The grey bars correspond to inside Coliumo Bay (E2, E3, E6), and the black bars correspond to outside the bay (E7 and E4). There was no pigment data for January 18th. (DOCX) [file pone.0179023.s004.docx]

**Supporting Information (S4 Fig)**
