# Supplementary material for: Short-term alteration of biotic and abiotic components of the pelagic system in a shallow bay produced by a strong natural hypoxia event
Source: PLoS One. 2017 Jul 17;12(7):e0179023. doi: 10.1371/journal.pone.0179023 (PMC5513412; doi:10.1371/journal.pone.0179023)
Supplement: S5 Fig — Average (±SD) abundance (cells mL-1) of total nanoplankton in Coliumo Bay from January 3rd, to 18th, 2008 (a) autotrophic nanoflagellates (ANF), (b) heterotrophic nanoflagellates (HNF), (c) dinoflagellates. The grey bars correspond to inside Coliumo Bay (E2, E3, E6), and the black bars correspond to outside the bay (E7 and E4). (DOCX) [file pone.0179023.s005.docx]

**Supporting Information (S5 Fig)**
