# Supplementary material for: Short-term alteration of biotic and abiotic components of the pelagic system in a shallow bay produced by a strong natural hypoxia event
Source: PLoS One. 2017 Jul 17;12(7):e0179023. doi: 10.1371/journal.pone.0179023 (PMC5513412; doi:10.1371/journal.pone.0179023)
Supplement: S6 Fig — The first and second component explained 37% and 33% of the total variance, respectively. Environmental variables are surface (-S) and bottom (-b) temperature (T), salinity (Sal), dissolved oxygen (Ox), pH, and redox (R). Average water column nutrients (Nitrate, nitrite, phosphate, silicic acid) and pigments (Chlorophyll a, phaeopigments) were also used in the analysis. (DOCX) [file pone.0179023.s006.docx]

**Supporting Information (S6 Fig)**
