# Supplementary material for: Short-term alteration of biotic and abiotic components of the pelagic system in a shallow bay produced by a strong natural hypoxia event
Source: PLoS One. 2017 Jul 17;12(7):e0179023. doi: 10.1371/journal.pone.0179023 (PMC5513412; doi:10.1371/journal.pone.0179023)
Supplement: S1 Table — (DOCX) [file pone.0179023.s007.docx]

**Supporting Information (S1 Table)**

**S1 Table.** Summary of the micro-phytoplankton taxa in Coliumo Bay from January 3rd, to 18th, 2008 Numbers in brackets indicate relative abundance as a function of total living and dead cells: 1) >5%-<10% of dead cells, 2) >10% of dead cells, 3) >5%-<10% of living cells, 4) >10%-<15% of living cells, 5) > 15% of living cells.

| **Diatoms** | **Dinoflagellates** | **Cyanobacteria** | **Ciliates** | **Chlorophyceae** | **Coccolithophores** |
| --- | --- | --- | --- | --- | --- |
| *Actinocyclus curvatus* | *Gymnodinium* sp. | *Oscillatoria submembranacea* | *Helicostomella* sp. | *Scenedesmus acuminates* | Coccolithophore sp. |
| *Amphipheura* sp. | *Gyrodinium* sp. (5) | *Schizothrix calcicola* (2,3) | *Strobilidium* sp. |  |  |
| *Amphora* sp. | *Gonyaulax* sp. | *Oscillaria* sp. |  |  |  |
| *Chaetoceros* sp. (3) | *Protoperidinium* sp. (4) | *Oscillatoria submembranacea* (4) |  |  |  |
| *Chaetoceros socialis* (1,5) | *Prorocentrum* sp. | *Anabaena* sp. |  |  |  |
| *Chaetoceros contrictus* | *Oxytoxum* sp. |  |  |  |  |
| *Chaetoceros debilis* (3) |  |  |  |  |  |
| *Chaetoceros* (cists) (2,3) |  |  |  |  |  |
| *Cocconeis* sp. |  |  |  |  |  |
| *Coscinodiscus* sp. |  |  |  |  |  |
| *Cylindrotheca closterium* |  |  |  |  |  |
| *Cylindrotheca longissima* |  |  |  |  |  |
| *Detonula* sp. |  |  |  |  |  |
| *Fragilaria* sp.(1) |  |  |  |  |  |
| *Fragilariopsis* sp.(1) |  |  |  |  |  |
| *Gomphonema* sp. |  |  |  |  |  |
| *Grammatophora* sp. |  |  |  |  |  |
| *Gyrosigma fasciola* |  |  |  |  |  |
| *Licmophora abbreviata* (1) |  |  |  |  |  |
| *Navicula ammophila* |  |  |  |  |  |
| *Navicula delicatula* (1) |  |  |  |  |  |
| *Navicula dicephala* |  |  |  |  |  |
| *Navicula* sp. |  |  |  |  |  |
| *Navicula directa* |  |  |  |  |  |
| *Odontella longicrucis* |  |  |  |  |  |
| *Odontella aurita* |  |  |  |  |  |
| *Pleurosigma* sp. |  |  |  |  |  |
| *Pleurosigma normanii* |  |  |  |  |  |
| *Pseudonitzschia seriata* (1) |  |  |  |  |  |
| *Nitzschia paradoxa* |  |  |  |  |  |
| *Pseudo-nitzschia delicatissima* |  |  |  |  |  |
| *Pseudo-nitzschia* sp. |  |  |  |  |  |
| *Thalassionema* sp. |  |  |  |  |  |
| *Thalassiosira* sp.(1,3) |  |  |  |  |  |
| *Rhabdonema* sp. |  |  |  |  |  |
| *Rhoicosphenia curvata* |  |  |  |  |  |
| *Leptocylindrus danicus* |  |  |  |  |  |
| *Synedra* sp. |  |  |  |  |  |
