# Supplementary material for: Short-term alteration of biotic and abiotic components of the pelagic system in a shallow bay produced by a strong natural hypoxia event
Source: PLoS One. 2017 Jul 17;12(7):e0179023. doi: 10.1371/journal.pone.0179023 (PMC5513412; doi:10.1371/journal.pone.0179023)
Supplement: S2 Table — (DOCX) [file pone.0179023.s008.docx]

**Supporting Information (S2 Table)**

**S2 Table.** Results, of a PLS regression for macro-zooplankton, micro-phytoplankton, and nanoplankton, and density as an environmental function variable. (a) PLS loadings for each PLS component. (b) PLS scores in the first and second PLS components. r^2^ = proportion of the variance accounted for the PLS regression. *p* values lower than 0.05 are in bold, and indicate a significant result. Phaep = phaeopigments, Chl *a* = chlorophyll *a*, T-s = surface temperature, T-b = bottom temperature, Sal-s = surface salinity, Sal-b = bottom salinity, Ox-s = surface dissolved oxygen, Ox-b = bottom dissolved oxygen, Redox-s = surface redox potential, and R-b = bottom redox potential.

(a)

| **Independent Variable** | **Component 1** | **Component 2** |
| --- | --- | --- |
| Log Macrozooplankton Density | 0.093 | 0.533 |
| Log Living Microphytoplankton Density | -0.306 | -0.278 |
| Log Nanoplankton Density | -0.319 | 0.194 |

(b)

| **Independent Variable** |  | |
| --- | --- | --- |
|  | Log Macrozooplankton Density | |
|  | Log Living Microphytoplankton Density | |
|  | Log Nanoplankton Density | |
| **Dependent Variable** |  |  |
|  | **Component 1** | **Component 2** |
| Phaep (mg m^-3^) | 0.369 | 0.127 |
| Chl *a* (mg m^-3^) | -0.222 | 0.388 |
| Nitrite (μM) | 0.371 | -0.197 |
| Nitrate (μM) | -0.328 | -0.240 |
| Phosphate (μM) | -0.309 | -0.320 |
| Silicic Acid (μM) | -0.169 | 0.471 |
| T-s (°C) | -0.081 | -0.042 |
| T-b (°C) | 0.105 | 0.439 |
| Sal-s | 0.073 | 0.135 |
| Sal-b | -0.133 | 0.178 |
| Ox-s (mL L^-1^) | -0.386 | 0.052 |
| Ox-b (mL L^-1^) | -0.294 | 0.258 |
| pH-s | -0.337 | -0.050 |
| pH-b | -0.376 | 0.224 |
| Redox-s (mV) | -0.079 | 0.156 |
| Redox-b (mV) | -0.211 | 0.240 |
| **r^2^** | **0.37** | **0.33** |
| ***P*** | **0.036** | |
