# Supplementary material for: Short-term alteration of biotic and abiotic components of the pelagic system in a shallow bay produced by a strong natural hypoxia event
Source: PLoS One. 2017 Jul 17;12(7):e0179023. doi: 10.1371/journal.pone.0179023 (PMC5513412; doi:10.1371/journal.pone.0179023)
Supplement: S3 Table — (DOCX) [file pone.0179023.s009.docx]

**Supporting Information (S3 Table)**

**S3 Table.** Spearman correlations (r_S_) between the most abundant macro-zooplankton taxa and concentration of surface dissolved oxygen during the hypoxia event and for the quarterly samples from January 2007 and January 2009. Macro-zooplankton data were transformed to log x+1. N is the sample size; values of p < 0.05 are indicated in bold.

|  | **r** | **N** | ***p*** |
| --- | --- | --- | --- |
| **Total macrozooplankton** | 0.608 | 40 | **<0.001** |
| **Copepoda** |  |  |  |
| *Acartia* *tonsa* | 0.747 | 40 | **<0.001** |
| *Calanus patagoniensis* | 0.351 | 40 | **0.027** |
| *Calanus chilensis* | 0.641 | 40 | **<0.001** |
| *Paracalanus* sp. | 0.540 | 40 | **<0.001** |
| *Rhyncalanus* sp. | -0.103 | 40 | 0.526 |
| *Centropages* sp. | 0.378 | 40 | **0.016** |
| *Oithona* sp. | 0.222 | 40 | 0.168 |
| Harpacticoidea | -0.375 | 40 | **0.017** |
|  |  |  |  |
| **Other crustaceans** |  |  |  |
| Cumacea | 0.183 | 40 | 0.258 |
| Isopoda | 0.199 | 40 | 0.219 |
| Amphipoda | -0.539 | 40 | **<0.001** |
| Mysidacea (*Neomysis* sp.) | 0.025 | 40 | 0.877 |
| Euphausiacea | 0.281 | 40 | 0.080 |
|  |  |  |  |
| **Gelatinous organisms** |  |  |  |
| Larvacea | 0.411 | 40 | **0.008** |
| Chaetognatha | -0.001 | 40 | 0.998 |
| Ctenophora | -0.017 | 40 | 0.919 |
| Medusae | 0.184 | 40 | 0.256 |
| Siphonophora | 0.210 | 40 | 0.193 |
|  |  |  |  |
| **Larvae and eggs** |  |  |  |
| Polychaeta larvae | 0.288 | 40 | 0.071 |
| Mollusca larvae | 0.306 | 40 | **0.055** |
| Zoea larvae | 0.669 | 40 | **<0.001** |
| Megalopa larvae | -0.026 | 40 | 0.872 |
| Crustacean eggs | -0.589 | 40 | **<0.001** |
| Fish larvae | 0.250 | 40 | 0.119 |
| Fish eggs | 0.374 | 40 | **0.018** |
| Cypris larvae | 0.421 | 40 | **0.007** |
| Mysis larvae | 0.480 | 40 | **0.002** |
| Alima larvae | 0.396 | 40 | **0.011** |
