# Supplementary material for: Short-term alteration of biotic and abiotic components of the pelagic system in a shallow bay produced by a strong natural hypoxia event
Source: PLoS One. 2017 Jul 17;12(7):e0179023. doi: 10.1371/journal.pone.0179023 (PMC5513412; doi:10.1371/journal.pone.0179023)
Supplement: S4 Table — (a) PERMDISP output for the analyses conducted for environmental and biological variables in Coliumo Bay. (b) PERMDISP pair-wise output for macrozooplankton inside Coliumo Bay. (DOCX) [file pone.0179023.s010.docx]

**Supporting Information (S4 Table)**

**S4 Table.** (a) PERMDISP output for the analyses conducted for environmental and biological variables in Coliumo Bay. Factor refers to similar categories used in the PERMANOVA analyses. Resemblance analysis was conducted in a similar manner to the PERMANOVA analyses. (b) PERMDISP *pair-wise* output for macro-zooplankton inside Coliumo Bay from January 2007 to January 2009, including January 3^rd^, to 18^th^, 2008. In bold *p* values < 0.01.

| **(a) Variable** | **Factors** | ***F*** | **df** | ***P*(perm)** |
| --- | --- | --- | --- | --- |
|  |  |  |  |  |
| Water column | Sampling period | 6.4409 | 1,21 | 0.0158 |
|  | Zone | 2.4942 | 1,21 | 0.1630 |
| Compositional density of total microphytoplankton | Sampling period | 0.7968 | 1,19 | 0.4230 |
|  | Zone | 0.1965 | 1,19 | 0.7096 |
| Presence-absence of total microphytoplankton | Sampling period | 0.9123 | 1,19 | 0.4032 |
|  | Zone | 0.7723 | 1,19 | 0.4854 |
| Compositional density of live microphytoplankton | Sampling period | 1.3598 | 1,19 | 0.2998 |
|  | Zone | 0.0674 | 1,19 | 0.8311 |
| Presence-absence of live microphytoplankton | Sampling period | 5.3424 | 1,19 | 0.0538 |
|  | Zone | 0.3132 | 1,19 | 0.6476 |
| Macrozooplankton inside Coliumo Bay | Sampling period | 12.392 | 2,74 | **0.0004** |
|  |  |  |  |  |
| **(b) Variable** | **Groups** | **t** |  | ***P*(perm)** |
| Macrozooplankton inside Coliumo Bay | Before, After | 5.8665 |  | **0.0001** |
|  | Before, During | 2.4665 |  | 0.0354 |
|  | During, After | 0.2561 |  | 0.8516 |
