# Supplementary material for: Short-term alteration of biotic and abiotic components of the pelagic system in a shallow bay produced by a strong natural hypoxia event
Source: PLoS One. 2017 Jul 17;12(7):e0179023. doi: 10.1371/journal.pone.0179023 (PMC5513412; doi:10.1371/journal.pone.0179023)
Supplement: S5 Table — (DOCX) [file pone.0179023.s011.docx]

**Supporting Information (S5 Table)**

**S5 Table.** PERMANOVA *pair-wise* output for macro-zooplankton inside Coliumo Bay from January 2007 to January 2009, including January 3^rd^, to 18^th^, 2008. (Groups) refers to each sampling period (Before, during, and after hypoxia event). In bold *p* values < 0.05. Resemblance analysis was obtained with Jaccard similarity coefficient and presence-absence data.

| **Groups** | **t** | ***P*(perm)** | **Unique perms** |
| --- | --- | --- | --- |
| Before, After | 2.3002 | **0.0001** | 9942 |
| Before, During | 4.1521 | **0.0001** | 9923 |
| During, After | 3.3365 | **0.0001** | 9939 |
